# Health economic analysis of organizational models for breast cancer surgery: a bottom-up micro-costing and cost-minimization approach

**DOI:** 10.1186/s13561-026-00743-x

**Published:** 2026-02-12

**Authors:** Johan Eriksson, Kaspar Walter Meili, Lena Lindholm, Micael Appelblad, Malin Sund

**Affiliations:** 1 Department of Diagnostics and Intervention/Surgery, Umeå University, Umeå, Sweden; 2 Department of Epidemiology and Global Health, Umeå University, Umeå, Sweden; 3 Heart center, University Hospital of Norrland, Region Västerbotten, Umeå, Sweden; 4 Department of Public Health and Clinical Medicine, Umeå University, Umeå, Sweden; 5 Department of Surgery, University of Helsinki and Helsinki University Hospital, Helsinki, Finland

**Keywords:** Breast cancer surgery, Micro-costing, Cost-minimization, Waiting times, Healthcare resource allocation

## Abstract

**Background:**

Healthcare systems face challenges in optimizing resources while maintaining high-quality care. Breast cancer surgery represents a substantial share of elective surgery and provides an opportunity to evaluate different organizational models. This study presents a health economic analysis comparing two models for breast cancer surgery at the same hospital.

**Methods:**

A bottom-up micro-costing approach was employed to evaluate potential cost-savings of breast cancer surgeries performed at a general surgical department (GS) versus a cardiothoracic surgery department (CT). We analyzed 543 consecutive patients undergoing elective breast cancer surgery between January 2014 and September 2016. Resource use was identified through direct observation, hospital administrative systems, and operating room logs. Personnel, disposables, medications, and facility costs were quantified based on observed resource use within the study dataset; no external benchmarking was performed.

**Results:**

CT was less expensive, with an average saving of 3,547 Swedish krona (SEK) per operation (95% CI: -674 to 7,510 SEK). Bootstrap analysis with 1,000 iterations showed CT was less costly in 96.2% of samples. Procedures were shorter at CT (170.8 vs. 221.3 min), enabling more operations per day (3.2 vs. 2.4). In our deterministic simulation, removing CT capacity increased waiting times by 15%, from 39 to 45.1 days, conditional on steady inflow and constant OR availability. Annual savings at the observed annual volume (~ 192 patients) were 681,104 SEK and could reach ~ 1.77 million SEK if volumes increased to 500 patients/year.

**Conclusions:**

The CT organizational model was more likely to be less costly while maintaining shorter waiting times. These findings suggest that CT capacity may be prioritized, particularly at higher patient volumes, to support both economic efficiency and patient access.

**Supplementary Information:**

The online version contains supplementary material available at 10.1186/s13561-026-00743-x.

## Introduction

Healthcare systems in many countries face increasing pressure to optimize resource utilization while maintaining high-quality care. This challenge has been exacerbated by the COVID-19 pandemic, which has created substantial backlogs of surgical cases [1–4], with the risk of increased morbidity and mortality among waiting patients [5–7].

Over recent years, there has been growing emphasis on reducing time to surgery (TTS) for breast cancer patients [8–10]. Several studies have found significant relationships between waiting time and adverse disease outcomes, potentially including disease progression and survival [11–13]. Additionally, longer pre-operative waiting times can increase patient anxiety and decrease satisfaction with care [14].

While additional resources for cancer surgery have been advocated [15], optimizing organizational aspects of care delivery may be a more sustainable approach. Various cost-saving strategies in surgery have been explored, including standardization and streamlining [16, 17], surgeon scorecards [18], and operating room scheduling optimization [19–21]. Such initiatives share conceptual similarities with standardized care pathways, such as Sweden’s ‘standardiserade vårdförlopp’ (SVF), aiming to improve efficiency and consistency in care delivery. Less attention has, however, been paid to how the organizational model itself affects both costs and clinical outcomes.

Accurate cost assessment is critical for meaningful economic evaluations of healthcare interventions. Traditional “top-down” or “gross-costing” approaches use average costs to estimate resource use, such as healthcare resource groups (HRGs) or diagnosis-related groups (DRGs). While straightforward, this method is often too crude to compare specific interventions or modifications to existing procedures [22]. Micro-costing, defined as the “direct enumeration and costing of every input consumed in the treatment of a particular patient” [23], provides a more precise assessment of economic costs [24].

This study is a follow up analysis that builds upon findings from a previous study [25], where a multivariate analysis identified a significant time difference of 30.67 min per operation between departments, with the Cardiothoracic surgery department (CT) being faster than the general surgical department (GS) after adjusting for patient characteristics (age, BMI, ASA class), procedure category, and the presence of trainee surgeons. At the study site the same set of surgeons operated on breast cancer patients at the CT and GS departments and patients were allocated to surgery at either department without selection.

In our setting, the comparison is not between different surgical techniques but between two organizational models delivering the same elective breast cancer surgery by the same set of surgeons within the same hospital. Patients follow identical pre- and postoperative care pathways and perioperative protocols; the operating department is the only organizational difference. This creates a relevant decision problem for hospital management: whether allocating intermittent operating room capacity to an alternative unit can reduce perioperative resource use and improve access (waiting time) without changing the clinical care pathway. Therefore, we evaluate perioperative costs and capacity implications within a cost-minimization framework, acknowledging that postoperative outcomes are not available in our dataset and should be examined in future work.

This study aims to conduct a detailed health economic analysis of two organizational models for breast cancer surgery at the same hospital: departments GS and CT. By using a rigorous bottom-up micro-costing methodology, we compare the economic factors between these two units while also assessing capacity aspects and waiting times to provide comprehensive decision support.

## Methods

### Methodological Approach

For this health economic analysis, we used a bottom-up micro-costing approach within a cost-minimization framework, assuming equivalent clinical outcomes between the two departments. Bottom-up micro-costing is considered a precise method for comparing costs between organizational models in healthcare [26, 27]. We identified and valued resource use at the case level for the perioperative episode based on operating-room logs, direct observations, and hospital accounting data, and expressed all costs in the selected price year. This approach is particularly useful in surgical care where high-cost consumables or implants may contribute substantially to total costs [28].

### Study Design and Cost Components

#### System boundaries and study population

The analysis focused on the perioperative phase of breast cancer surgery in two operating departments (GS and CT) at the same hospital (a university hospital in Sweden). Sweden operates a tax-funded public healthcare system with centralized cancer care, where all breast cancer surgery in the hospital’s catchment area is performed at this single tertiary center, ensuring a representative and unselected patient population.

The study population consisted of 543 consecutive patients who underwent elective breast cancer surgery between January 2014 and September 2016, distributed between GS (*n* = 445) and CT (*n* = 98).

The CT unit is primarily a cardiothoracic operating department and does not provide breast cancer surgery in its regular case-mix. Instead, CT contributed intermittent “external” OR capacity to the hospital’s breast- and endocrine surgery unit when the cardiothoracic list pressure was low and room-days could be released without compromising CT’s core activity. Decisions to release such capacity were made ad hoc during periodic OR planning reviews (typically several times per year), resulting in variable availability over time (reflected in our seasonal activity index). Importantly, allocation of individual patients to GS versus CT was not based on clinical selection; cases were scheduled from a common breast surgery waiting list primarily according to the first available OR slot. Patients followed identical pre- and postoperative care pathways within the same hospital, and the same surgeon group operated in both settings; the operating department was the only organizational difference. We included consecutive elective operations scheduled through the hospital breast surgery pathway during January 2014–September 2016. Eligible procedures comprised breast-conserving surgery and mastectomy, with or without sentinel node biopsy/axillary lymph node dissection, as well as other elective breast procedures handled within the same surgical program (classified as “limited” vs. “extensive”; definitions below). Emergency operations and cases not managed within the breast surgery pathway were not eligible. “Extensive procedures” were identified using CCM together with diagnosis C50.* and including modified radical mastectomy (HAC22), resection of breast tissue with axillary lymph node dissection/axillary procedures (HAB** + PJD42), and axillary lymph node dissection (PJD42). “Limited procedures” were identified using CCM together with diagnosis C50.* and including resection of breast tissue with or without sentinel node biopsy (HAB** ± PJA10), simple mastectomy with or without sentinel node biopsy (HAC** ± PJA10), local excision (HAF**), biopsy/incision (HAA10), minor corrections (HAD99), and selected reoperation codes (HWA00/HWC00/HWE00/HWD00/HWW99). For group allocation, at least two of the listed codes were required (e.g., C50.1 + PJD42 for extensive procedures; C50.1 + HAB20 ± PJA10 for limited procedures). “Consultant present” indicates that the main surgeon was a consultant. “High-volume surgeon” was defined as a main surgeon performing > 50 procedures per year.

#### Cost categories

The cost analysis included direct personnel costs for all operating room staff, including surgeons (specialists and residents/trainees), anesthesiologists, anesthesia nurses, operating room nurses, and nursing assistants, as well as costs for medications used during anesthesia and surgery, and facility costs related to operating room usage. All costs were calculated based on observed resource use within the study dataset, and no external benchmarking was performed.

#### Costing perspective and price year

We conducted a bottom-up micro-costing from the provider (hospital) perspective over the perioperative episode. Our approach captures the dominant time-driven cost mechanisms using case-level timestamps and observed staffing. Resource use (staff minutes by profession, medications, consumables, and room time) was measured from 2014 to 2016 operating logs and direct observations. Unit costs were valued using 2025 price data, i.e. payroll (including social security and vacation benefits) from the hospital’s 2025 payroll system, medication prices from the 2025 regional formulary/procurement lists, and facility costs from the 2025 internal rent and allocation schedules. Facility costs represent an all-inclusive internal OR cost rate used by the hospital for costing and internal charging (covering premises and overhead components). All costs are reported in 2025 Swedish krona (SEK). We therefore combined 2014–2016 quantities with 2025 unit prices (replacement costing) to reflect the current cost level and avoid additional indexation assumptions. No external benchmarking was applied. Because a single price year was used for valuation, no additional inflation adjustment or discounting was required.

#### Exclusion criteria

Preoperative and postoperative costs were excluded from the analysis, as were hospital-level overhead (assumed equal between departments) and long-term follow-up costs. Additionally, patient cases with incomplete data regarding waiting times or operation reports were excluded from the capacity analysis.

### Data Collection

#### Time definitions

In this study we distinguish between the following time concepts:

- Preoperative waiting time (queue): Interval before the day of surgery (from decision/eligibility to the scheduled operation date). Not part of operating-room case time.
- Preparation time (room setup): Day-of-surgery tasks to prepare the operating room and equipment before incision. In our data, aggregated preparation/clean-up time was split 50/50 around the procedure boundaries for analysis.
- Intraoperative waiting time (operating room idle time): Non–value-adding time on the day of surgery when the operating room and team are ready, but the patient has not yet arrived in the room.
- Procedure time: From incision to completion of the operation, when the surgeon is present and actively operating.
- Post-procedure clean-up: Day-of-surgery tasks to close down the room after the operation.
- Case time: Sum of day-of-surgery components: preparation time + intraoperative waiting time + procedure time + post-procedure clean-up. (Preoperative waiting time is not included in case time.)

**Fig. 1:**
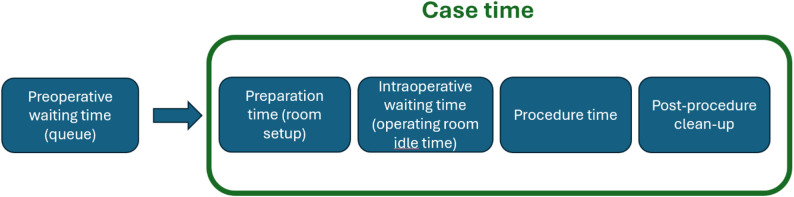
– Conceptual schematic of time definitions

A conceptual schematic of the time definitions is shown in Fig. 1.

Figure 1. Components of the patient pathway and operating room time definitions. Preoperative waiting time refers to the queue time from being listed to the day of surgery and is modeled separately from operating room case time. Case time comprises (i) preparation time (room set-up and patient preparation), (ii) intraoperative waiting/idle time (operating room idle time between steps), (iii) procedure time (knife-to-skin to closure), and (iv) post-procedure clean-up (turnover/room reset). These components correspond to timestamps extracted from operating logs and direct observations.

#### Time measurements

Time measurements were extracted from the Orbit4 (Tietoevry Lifecare, Espoo, Finland) electronic anesthesia record and operation reports and grouped into: (i) preparation and clean-up time, (ii) intraoperative waiting time (operating room idle time), and (iii) procedure time. Preparation and clean-up time comprised pre- and post-procedure tasks and was split evenly around the procedure boundaries (50% before incision start and 50% after procedure end). Intraoperative waiting time (operating room idle time) was defined as the period on the day of surgery after all preparations were complete, and the operating room and team were ready, until the patient arrived in the operating room. Procedure time covered the interval from incision to completion of the operation, when the surgeon was present and actively operating. These components were summed to calculate the total case time. This definition of intraoperative waiting time differs from the preoperative waiting times (the queue before the surgical date) analyzed separately later in the study.

#### Personnel resources and presence

Personnel resource usage was documented through systematic observation of 109 operations (69 at GS and 40 at CT). We registered the presence and time in-room for all operating-room staff categories (surgeons, anesthesiologists, anesthesia nurses, OR nurses, and nursing assistants). Anesthesiologist presence was additionally quantified as a proportion of total case time because it differed structurally between departments. Anesthesiologist presence at GS averaged 40% (95% CI: 37%–43%) during preparation and post-procedure work, while presence at CT was 100%.

#### Salary data

Salary costs were obtained from the Region’s payroll system for each personnel category and included social security contributions and vacation compensation.

#### Medication and facility costs

Individual medication doses per case were not available in the operating logs. Medication consumption was therefore estimated using a phase-based dosing assumption: a fixed induction component and time-proportional maintenance during anesthesia (assumed during all procedure time and a defined share of preparation and post-procedure time), valued using 2025 formulary prices. Medication costs were calculated based on estimated usage according to the different phases of the operation, with induction time standardized to 5 min per operation to reflect the average duration of anesthesia induction and ensure consistent cost estimation. Prices were obtained from regional pharmaceutical supply, and dosing according to protocols. Facility costs were based on the hospital controller’s internal OR cost rate (“hourly rent”), an all-inclusive overhead rate used for internal costing and charging that covers premises and indirect cost components (e.g., depreciation, utilities/energy, maintenance, and support/overhead services).

### Calculation Methods

### Personnel costs

Personnel costs were calculated using the following formula:$$\begin{aligned}&\:Cost\:per\:profession\\&=\:working\:time\:in\:minutes\:\\&\times\:\:minute\:salary\:\times\:\:number\:of\:staff\:\\&\times\:\:presence\:factor\end{aligned}$$    

Working time per case was partitioned according to the components defined in Time definitions (preparation and clean-up, intraoperative waiting and procedure time).

### Medication and facility costs

#### Medication costs were calculated as

$$\:Total\:medication\:cost\:=\:\varSigma\:\:(Dosage\:\times\:\:Unit\:price)\:for\:all\:medications$$

#### Facility costs were calculated as

$$\:Facility\:cost\:per\:operation\:=\:hourly\:rent\:\times\:\:operation\:time\:in\:hours$$

#### Bootstrapping

Uncertainty in cost differences, both total and component-level, was quantified using a grouped, case-level non-parametric bootstrap, resampling cases within department (CT, GS) with replacement while preserving group sizes. We ran 1,000 replications and report percentile-based 95% confidence intervals and Pr(GS > CT). Full details are provided under Statistical analysis, subheading Inference for cost differences (bootstrap).

### Statistical analysis

All statistical analyses were performed with R version 4.5.0 (2025-04-11 ucrt).

#### Cost data

Cost data were summarized using means, medians, and standard deviations. Between-department uncertainty and contrasts were evaluated with the grouped bootstrap described below; t-tests are reported as descriptive complements only.

#### Modeling of capacity and waiting time

To estimate the impact of CT capacity on surgical waiting times, we used a deterministic simulation model based on observed patient flow and average operation times from the study dataset. The model assumed a steady weekly inflow of patients and constant operating room availability. Average waiting times were calculated by comparing scenarios with and without CT capacity under current patient volumes. Capacity was derived as: Procedures/day = 540 min/mean total case time. The weekly inflow was estimated from the observed surgical volume during the study period (total number of cases divided by number of study weeks). Waiting time scenarios contrasted ‘CT on’ vs. ‘CT off’. Model verification consisted of checking that the baseline (‘CT on’) scenario reproduced the observed mean preoperative waiting time among cases with complete time-to-surgery data. In the “CT off” scenario, CT room-days were removed from the system while GS OR availability was held constant, and the same steady inflow was assumed, yielding higher queueing and mean waiting time.

#### Inference for cost differences (bootstrap)

We quantified uncertainty in costs using a grouped, case-level non-parametric bootstrap. Cases were resampled within department (CT and GS) with replacement, preserving group sizes at each replication. We ran 1,000 replications with a fixed random seed. The primary contrast was the mean cost difference defined as GS minus CT. We report percentile-based 95% confidence intervals and Pr(GS > CT), the proportion of replications with GS − CT > 0. (With *R* = 1,000, Monte Carlo error for probabilities around 0.96 is ≈ 0.5–0.7% points.) The same resampling scheme was applied to component-level cost differences and their shares of the total difference.

#### Sensitivity analysis

To test result robustness, sensitivity analyses were performed by varying anesthesiologist presence (0%, 40%, 50%, 100%). The 40% level represents our empirical finding from sampling operations at GS, which showed an average presence of 40% (95% CI: 37%–43%) during preparation and post-procedure work. We included 0% and 100% as theoretical minimum and maximum values, with 50% as an additional intermediate value, to comprehensively assess the impact of this organizational difference on overall costs. Anesthesiologist presence was chosen for sensitivity testing because it contributes substantially to the overall cost difference between departments and represents the most uncertain parameter in the analysis. Other major cost components, such as medication prices and facility costs, were based on standardized regional data and remained constant between departments, making them less relevant for sensitivity testing as they only vary indirectly with operation time.

#### Data quality and validation

To ensure data quality, approximately 40% of the operations were manually verified against the medical records, including procedure times and staffing data. Additionally, potential outliers in total cost were assessed using Tukey’s method for boxplots. This identified one outlier, which was manually reviewed and found to be correctly entered.

#### Capacity and waiting time analysis

In addition to the cost analysis, two supplementary analyses were performed. The capacity analysis involved calculating operation capacity per room-day for each department, while the preoperative waiting time comparative analysis examined waiting time distributions between departments and assessed the CT department’s contribution to overall capacity.

For the preoperative waiting time analysis, data from patients with complete waiting time information were utilized, comprising 98 patients for CT and 445 patients for GS, representing the complete dataset available for comparative analysis. Patients were allocated from a common waiting list without systematic selection to either department, ensuring comparable handling between the two groups. This analysis encompassed descriptive statistics and distribution analysis of waiting times by department, assessment of waiting time variability and identification of outliers, and capacity utilization calculations based on observed operation times and throughput.

#### Seasonal analysis

To explore seasonal variation in CT usage, we calculated a CT activity index defined as the ratio between the proportion of all CT operations performed in each quarter and the proportion expected if CT operations were evenly distributed across all quarters. An index > 1 indicates overrepresentation, whereas an index < 1 indicates underrepresentation.

Outcomes.

Primary outcome was mean perioperative cost per case. Secondary outcomes were total case time, capacity (procedures per OR day), and preoperative waiting time. We assumed comparable clinical outcomes between departments because surgery was performed at the same hospital under the same clinical pathways and perioperative protocols, with patients admitted to the same wards pre- and postoperatively; the only difference was the operating department, and the same surgeon group operated in both settings. As our dataset did not contain postoperative complications or downstream resource use, we limited the economic evaluation to the perioperative episode and interpreted results as a cost-minimization analysis conditional on comparable outcomes. We highlight this as a limitation and suggest that future studies link postoperative outcomes and follow-up resource use to operating unit to empirically verify this assumption. Reporting followed CHEERS 2022 [29].

## Results

### Study population

The study cohort comprised 543 consecutive elective cases performed between January 2014 and September 2016, with 445 procedures conducted in the General Surgery department (GS) and 98 in the Cardiothoracic Surgery department (CT). CT contributed intermittent “external” operating room capacity when cardiothoracic list pressure was low; therefore, CT volumes were variable over time and reflected ad hoc release of room-days rather than clinical selection. Baseline patient characteristics and procedure mix by department are summarized in Table 1.

**Table 1:** – Baseline characteristics of the study population (*n* = 543), by operating department. Values are mean (SD) or n (%)

Characteristic	Total (*n* = 543)	GS (*n* = 445)	CT (*n* = 98)
Age (years)	60.65 (15.32)	61.03 (15.17)	58.91 (15.93)
BMI	26.50 (4.83)	26.59 (4.86)	26.09 (4.69)
ASA class I	146 (26.9)	117 (26.3)	29 (29.6)
ASA class II	321 (59.1)	260 (58.4)	61 (62.2)
ASA class III	76 (14.0)	68 (15.3)	8 (8.1)
Male sex	70 (12.9)	61 (13.7)	9 (9.2)
Extensive breast cancer procedures	144 (26.5)	121 (27.2)	23 (23.5)
Limited breast cancer procedures	399 (73.5)	324 (72.8)	75 (76.5)
Consultant present	436 (80.3)	354 (79.5)	82 (83.7)
High-volume surgeon present	459 (84.5)	373 (83.8)	86 (87.7)

Baseline characteristics of the study population.

*Abbreviations*: *GS *General Surgery, *CT* Cardiothoracic Surgery. Extensive and limited procedure groups are defined in the Methods.

Cost Analysis.

The cost analysis showed that CT is less costly on average than GS, with an average cost saving of 3,547 SEK per operation (95% CI: -674-7,510 SEK). The bootstrap analysis with 1,000 iterations showed that CT was less costly in 96.2% of bootstrap samples.

The frequency distribution of cost differences from the bootstrap analysis showed a clear shift toward positive values, indicating that GS is consistently more expensive than CT. The highest frequency is observed in the range of 3,500-3,600 SEK, which was close to the calculated average difference of 3,547 SEK.

Results from the bootstrap analysis showed an average cost difference of 3,547 SEK with a 95% confidence interval ranging from − 674 SEK to 7,510 SEK. The probability that GS is more expensive was 96.2%. Sensitivity analyses confirmed that CT was consistently more cost-saving than GS under all reasonable assumptions regarding anesthesiologist presence, operation times, and personnel costs.

Cost components.

To illustrate cost drivers, we decomposed total costs into micro-costing components. Table 2 reports mean costs per case by component for CT and GS, the difference defined as GS minus CT, and each component’s share of the total difference. 95% CIs and Pr(GS > CT) were obtained from case-level, department-stratified non-parametric bootstrap. All amounts are in SEK.

**Table 2:** – Decomposition of total costs by component

Component	CT mean (SEK)	GS mean (SEK)	Diff (SEK)	Share of total diff (%)	CI 95% for diff (SEK)	Pr(GS > CT) (%)
Personnel	11,497	10,643	-853	-24.10	-2,502–1,074	15.30
Medications	22,353	26,742	4,389	123.70	1,807–7,107	100
Facility	41	53	12	0.30	7–18	100
Total	–	–	3,547	100	-674–7,510	96.20

Difference is defined as GS − CT (positive = GS more costly). “Share of total difference” is the component difference divided by the total difference (3,547 SEK). Shares can be > 100% or negative because components may offset each other. CIs and probabilities from case-level bootstrap stratified by department. Amounts rounded to whole SEK; percentages to two decimals.

### Capacity Analysis

#### Efficiency ratio and operation times

For each procedure, an efficiency ratio (GS TCT / CT TCT) was calculated to show the relationship between departmental efficiencies, where values greater than 1.0 indicate that CT performs the procedure faster than GS. See Table 3 below.

**Table 3:** – Comparison of operational efficiency, procedure times, and theoretical capacity between CT and GS departments

Parameter	GS	CT	Efficiency Ratio (GS/CT)
Mean total case time (min)	221.30	170.80	1.30
Operations per room-day (9 h)	2.40	3.20	0.75
Break-even capacity (room-days/week)	1.52	1.17	1.30

The efficiency ratio is calculated as GS total case time divided by CT total case time, and capacity per room-day assumes a 9-hour operating day.

For different procedures, the efficiency ratio between departments varied considerably. Mastectomy with sentinel node biopsy showed the most similar performance between departments (average ratio 1.26, range 0.60–2.56), while breast conserving surgery with sentinel node biopsy had a similar efficiency ratio of 1.26 (range 0.51–3.53). Mastectomy without an axillary procedure had an average efficiency ratio of 1.29 and showed the largest variation, with ratios ranging from 0.47 to 3.71, likely reflecting differences in case complexity and surgical approach. And, lastly, presumably benign breast tumor removal showed a moderate difference (average ratio 1.30, range 0.58–2.63).

Based on a 9-hour operating day (540 min), CT can theoretically complete 3.2 operations compared to 2.4 for GS per room-day. This efficiency difference becomes practically significant when planning weekly schedules across multiple operating rooms or when determining resource requirements for different patient volumes.

### Break-even Analysis

To keep pace with the current influx of 3.7 new patients per week, calculations indicate a need for 1.17 CT room-days per week. This demonstrates that CT’s superior efficiency allows the same patient volume to be handled with significantly fewer resources compared to the 1.52 room-days that would be required using the GS model.

#### Waiting time analysis and CT capacity significance

The CT department handled approximately 18% of the total operation volume (98 of 543 operations) during the study period. The distribution of operations between departments varied considerably between quarters, with a clear seasonal pattern. Mean knife-to-skin time was 45.08 min at GS and 39.04 min at CT. Given that operation time in this dataset serves primarily as a cost driver and is typically right-skewed, we present descriptive statistics rather than inferential tests here. Detailed inferential analysis of time differences between departments has been reported previously [25].

The simulation model indicates that removal of CT capacity would lead to a substantial increase in surgical waiting times. Removing CT capacity increased the mean preoperative waiting time from 39.0 to 45.1 days (+ 6.1 days; +15.6%), under current volumes and assuming constant OR availability.

#### Seasonal analysis of CT usage

The seasonal analysis shows that CT usage follows a clear seasonal pattern throughout the year, with a strong negative correlation with quarter number (*r*=-0.92). The CT activity index, representing the relative proportion of CT operations per quarter compared to an even distribution, indicates overrepresentation in Q3 (1.20) and underrepresentation in Q4 (0.71).

#### Economic consequences at different patient volumes

Based on the cost analysis, economic consequences were calculated for different patient volumes between only using CT vs. only using GS (see Table 4).

**Table 4:** – Projected annual cost savings using CT versus GS at different patient volumes

Patient volume (per year)	Annual cost savings (SEK)	95% CI (SEK)
Study cohort (*n* = 192)	681,104	-129,435–1,441,911
400	1,418,966	-269,656–3,003,981
500	1,773,707	-337,070–3,754,976

Values represent estimated savings based on mean perioperative costs per operation, with 95% confidence intervals calculated using bootstrap analysis.

#### Break-even capacity at different patient volumes

Break-even capacity, defined as the minimum number of operating room-days required to handle all incoming patients, was calculated for different annual patient volumes using mean operation times from the current study (CT: 170.8 min; GS: 221.3 min). The calculations assume a nine-hour operating day and constant room availability. These results represent theoretical capacity based on average procedure times; in practice, the number of operations per day is limited to whole patients, meaning up to three CT operations or two GS operations per room-day.

For an annual volume of 400 patients (approximately 7.7 patients per week), the theoretical break-even point corresponds to 2.43 CT room-days or 3.15 GS room-days per week. Under these assumptions, the most cost-efficient configuration is one CT room-day combined with two GS room-days, providing a total theoretical capacity of about 8.0 patients per week. Using three CT room-days alone would provide a theoretical capacity of 9.5 patients per week, whereas four GS room-days alone would accommodate approximately 9.8 patients per week.

For an annual volume of 500 patients (approximately 9.6 patients per week), the break-even point increases to 3.04 CT room-days or 3.94 GS room-days per week. At this higher patient volume, the most cost-efficient setup is three CT room-days, which would provide a theoretical capacity of 9.5 patients per week. Using four GS room-days alone would result in a capacity of around 9.8 patients per week, while a mixed setup of one CT and three GS room-days would provide capacity for approximately 10.5 patients per week.

Although three CT room-days fall slightly below the theoretical requirement at 500 patients per year (9.5 versus 9.6 patients per week), this difference of about 0.1 operations per week is operationally negligible. Allocating four CT room-days instead would create a substantial overcapacity, equivalent to about 12.6 patients per week.

## Discussion

This study presents a comprehensive health economic evaluation of two different organizational models for breast cancer surgery (CT and GS departments). As shown previously, the unique feature is that the same group of surgeons operated breast cancer patients at the two departments from a common waiting list without a specific selection of patients to either department [25]. The results clearly show that the CT department is less costly than GS, with an average cost saving of 3,547 SEK per operation. Because postoperative complications and downstream resource use were not available in our dataset, conclusions are conditional on comparable clinical outcomes between departments. If meaningful differences in postoperative morbidity or follow-up care existed, total costs could differ; future studies should link postoperative outcomes and downstream resource use to operating unit. This translates to a substantial annual savings potential of 681,104 SEK at current patient volume and could increase to approximately 1.77 million SEK if patient volumes were to reach around 500 patients per year. To capture uncertainty in cost estimates, we used bootstrap methods (see Statistical analysis). For operation times, we focused on procedure-specific analyses rather than pooled averages.

The lower perioperative cost of CT can be explained by several factors. A central difference is the lower average operation time, which substantially increases resource utilization efficiency. In our study, the mean total case time was 170.8 min at CT compared to 221.3 min at GS, corresponding to an average saving of 50.5 min per operation. This effect was even more pronounced for certain procedures, where some took more than twice as long at GS compared to CT. Furthermore, the analysis of anesthesiologist presence highlights clear organizational differences, with CT maintaining constant staffing while GS employs more variable presence. Importantly, the cost savings from shorter procedure times in the CT regime outweigh the higher personnel costs associated with the GS regime, underscoring the overall resource efficiency of the CT model.

The observed time/cost differences were primarily driven by organizational configuration in the anesthesia phase rather than different clinical task content. In our direct observations, CT consistently had a dedicated anesthesiologist present throughout the perioperative work, whereas GS showed more variable anesthesiologist presence during preparation and post-procedure work (mean 40%). This staffing configuration affects how anesthesia work is covered across phases and is one plausible driver of shorter total case time in the CT model. We did not conduct qualitative process mapping; therefore, we describe these mechanisms at the level of observed staffing patterns rather than attributing differences to specific behavioral or protocol factors. A plausible explanation is that CT’s anesthesia team structure enabled more parallel work and fewer handoffs during preparation and emergence. With a dedicated anesthesiologist present throughout the perioperative work, anesthesia-related tasks (e.g., induction/emergence decisions, troubleshooting, and coordination with the surgical team) could be handled without delays caused by competing responsibilities across rooms, potentially reducing idle time and smoothing transitions between phases. In contrast, more variable anesthesiologist presence at GS may increase the risk of micro-delays during critical transition points even when protocols are similar. We did not perform formal process mapping; these mechanisms should therefore be interpreted as hypotheses consistent with our observed staffing patterns and time differences.

It is particularly noteworthy that the results of this study align well with findings from a previous study [25], where a multivariate regression analysis – adjusting for patient age, BMI, ASA class, procedure category, and trainee presence – identified a significant time difference of 30.67 min per operation between departments. This consistency between two different methodological approaches – detailed micro-costing with direct observations in the present study, and statistical adjustment for confounders in that study – strengthens the evidence for a real efficiency difference between the organizational models.

The capacity analysis and waiting time simulation highlight the importance of the CT department in maintaining short preoperative waiting times. Our simulation indicates that waiting times would increase by 15% if CT capacity were removed, from 39 to 45.1 days. While this represents a modest but measurable impact on waiting times, the primary advantage of the CT department lies in its superior cost- and operational efficiency. Conversely, if the entire surgical volume could be handled with CT-level efficiency, waiting times might be reduced even further, particularly at higher patient volumes. The seasonal variation in CT usage observed in our analysis may reflect differences in patient volume, resource allocation, or other contextual factors, indicating that further investigation is warranted before drawing firm conclusions about optimization potential.

We did not analyze distributional effects or priority groups. As both organizational models maintained average waiting times well below national thresholds, we judge a low risk of unequal clinical consequences in this cohort. Future work should test whether waiting-time distributions differ by age, geography, and comorbidity.

The clinical significance of maintaining shorter preoperative waiting times extends beyond operational efficiency. Recent research has shown that waiting times exceeding 9 weeks (63 days) are associated with worse survival outcomes in breast cancer patients [30]. In our study, both organizational models maintained waiting times well below this threshold under current capacity conditions (39 days with CT, 45.1 days without CT). However, if CT capacity were removed and GS had to handle the entire patient volume, waiting times could eventually increase beyond acceptable levels, particularly at higher caseloads. The CT model’s ability to maintain shorter waiting times while simultaneously providing superior cost-savings therefore strengthens the argument for prioritizing this organizational approach.

It is important to note that while CT was more likely to be less costly than GS, this difference has less practical significance at current patient volumes. With an average volume of 3.7 patients per week, the unit can manage with two operating room-days per week at either department, or one room-day at each department, and still have excess capacity. This means that resource planning can consider factors other than perioperative cost alone, such as flexibility and availability.

With the adjusted times from a previous study [25], where CT was 30.67 min faster than GS (120.41 vs. 151.08 min), CT is even more competitive at higher patient volumes compared to the current micro-time study. For example, three CT room-days provide a theoretical capacity of 13.5 patients per week according to data from that study, which is more than sufficient to handle a volume of 500 patients per year (9.6 patients/week). This further strengthens the arguments for prioritizing CT capacity at higher volumes.

Our findings align with previous research in hospital micro-costing. Mercier and Naro [24] similarly found poor agreement between top-down and bottom-up costing methods in surgery services, highlighting the importance of detailed micro-costing for accurate resource assessment. They noted that bottom-up costing better captures the complexity of procedures and patient-level factors. Potter et al. also emphasized that micro-costing is particularly valuable for surgical interventions due to their resource-intensive nature, though they noted the need for standardization in methodology [22]. By enabling more efficient resource use, organizational models like CT could also help reduce surgical waiting times, potentially lowering the risk of disease progression and improving long-term outcomes for breast cancer patients.

### Strengths and Limitations

A strength of this study is the detailed bottom-up micro-costing methodology, which provides high precision in cost calculations. Through direct observations of 109 operations (69 at GS and 40 at CT), we were able to quantify resource use with high accuracy. Furthermore, the use of bootstrap methodology ensured robust confidence intervals despite potential skewness in the data. Cost estimates were then applied to the full study cohort and to projected patient volumes, meaning that extrapolation beyond the directly observed cases was based on standardized, systematically collected data.

An additional strength is that the results confirm and are supported by findings from a previous study on the same cohort [25], which, using a different methodological approach, also identified a significant time difference between departments. Compared to the adjusted times from that study, the present figures may underestimate the true time difference between CT and GS. This discrepancy likely reflects methodological differences between detailed micro-costing measurements and regression-adjusted estimates and should be considered when interpreting the results. The consistency across methods nevertheless strengthens the overall reliability of the conclusions.

A limitation is that the study focuses solely on the perioperative phase, meaning that potential differences in preoperative and postoperative costs are not captured. Furthermore, the study was conducted at a single hospital, which may limit the generalizability of the findings to other healthcare organizations with different structural conditions or patient populations.

Our costing approach is time-driven at the perioperative episode level and shares core principles with time-driven activity-based costing (TDABC). However, we did not undertake a full TDABC implementation, such as detailed process mapping across activities and estimation of practical-capacity cost rates for each resource, because the aim of this study was comparative episode-level micro-costing and capacity assessment of two organizational models within the same hospital rather than a process redesign/optimization project [31]. Moreover, the available routine data were sufficient to quantify case-level time and staffing for the perioperative episode, which are the dominant cost drivers in our decision problem.

Another limitation is that our capacity analysis used deterministic calculations based on average operation times and patient flow. Although allocation was not based on clinical selection, residual case-mix differences cannot be fully excluded. We also did not use propensity score matching/weighting; although baseline characteristics were similar, residual confounding is possible. Real-world patient arrivals typically follow stochastic patterns that may be better modelled using skewed distributions or queuing theory. However, because operating room arrivals are largely scheduled in advance, the impact of such stochastic variation is likely to be limited in this setting. Such approaches could provide more nuanced insights into capacity requirements during peak demand periods and support more robust resource planning strategies. Future studies could benefit from incorporating probabilistic models to account for variability in patient flow and operational uncertainties. We did not perform hypothesis testing for operation times in this dataset, as our primary inferential focus was on costs; instead, times are summarized descriptively and used as inputs to the cost model. This choice may limit formal inference on time differences within the present sample.

Generalizability is also limited by the single-hospital setting; transfer requires local adaptation of wages, internal rents, and case-mix. We address ethics and data protection elsewhere; equity aspects are discussed above.

Finally, certain data points were excluded from some analyses due to incomplete records, which may affect the precision of specific results. However, we maximized data utilization in each analytical step and verified that excluded cases did not differ systematically from those included cases.

## Conclusions

This comprehensive health economic analysis demonstrates that breast cancer surgery performed at the CT department is less costly on average than at GS, with an average saving of 3,547 SEK per operation. The CT department also contributes significantly to keeping waiting times down. At current patient volume, the annual savings potential amounts to 681,104 SEK, and at higher volumes, the savings could reach 1.77 million SEK annually.

Although CT was more likely to be less costly, this difference is of limited importance for resource planning at current patient volume because both departments have sufficient capacity with two room-days per week. At higher patient volumes, however, efficiency differences become more important, and our study suggests that CT-based solutions may be preferable at volumes of 400–500 patients per year. From a broader healthcare system perspective, resources saved through higher surgical efficiency could be reallocated to other areas of healthcare or public services. In addition, the findings suggest that improving GS efficiency towards CT levels could be an important policy objective.

In summary, this analysis provides a comprehensive decision basis showing that the CT organizational model is more cost-saving while also contributing significantly to keeping waiting times down. These results suggest CT capacity may be prioritized when outcomes are comparable and when CT capacity can be released without compromising core CT activity.

## Supplementary Information

Supplementary Material 1.

## Data Availability

Due to legal and privacy restrictions (GDPR), patient-level data cannot be shared publicly. Aggregated data and analytic outputs are available from the corresponding author on reasonable request. Custom code used for statistical analyses and simulations is available from the corresponding author on reasonable request. (Provide a repository link if preferred.)

## Abbreviations

CT: Cardiothoracic surgery
GS: General surgery
OR: Operating room
TTS: Time-to-surgery
SEK: Swedish krona
