# Supplementary material for: Health economic analysis of organizational models for breast cancer surgery: a bottom-up micro-costing and cost-minimization approach
Source: Health Econ Rev. 2026 Feb 12;16:24. doi: 10.1186/s13561-026-00743-x (PMC12930689; doi:10.1186/s13561-026-00743-x)
Supplement: Supplementary file 1 — Supplementary Material 1. [file 13561_2026_743_MOESM1_ESM.pdf]

Supplementary table S1

**Time component**	GS mean (min)	CT mean (min)	Diff (GS–CT) (min)
Room set-up (start → preparation complete)	71.30	52.10	19.20
Procedure (physician of record in → surgery finish)	97.79	84.59	13.20
Room clean-up (patient out → clean-up finished)	38.06	29.87	8.19
Subtotal (phase-defined components)	207.15	166.56	40.59
Intraoperative waiting/idle time (OR ready → patient in room)	14.15	4.24	9.91
Total case time (TCT)	221.30	170.80	50.50

Supplementary table S1: Phase-defined components (room set-up, procedure, clean-up) are taken from the phase definitions and mean values reported in our previous study [25] (definitions shown in the table). Total case time (TCT) is from the current manuscript (Table 3). Intraoperative waiting/idle time is measured as the interval from "preparation complete/OR ready" until "patient in room". Values are means; minor differences may occur due to rounding. Abbreviations: GS, general surgery; CT, cardiothoracic surgery; TCT, total case time; PTG, Procedural Time Glossary.
